# Supplementary material for: Association between Habitual Tea Consumption and Metabolic Syndrome and Its Components among Chinese Adults Aged 18~59 Years: Based on China Nutrition and Health Surveillance 2015–2017
Source: Nutrients. 2022 Aug 25;14(17):3502. doi: 10.3390/nu14173502 (PMC9459911; doi:10.3390/nu14173502)
Supplement: Supplementary file 1 [file nutrients-14-03502-s001.zip › Supplemental_final.pdf]

Table S1. Total energy intake, anthropometric index, and biomarkers of MetS among participants

Biomarkers*	Habitual tea consumption ($\bar{x}$±SD)				*p* for trend
	**None**	**1~2 cups/day**	**3~4 cups/day**	**Over 5 cups/day**	
E (Kcal/d)	2296.3±855.2	2463.6±879.4	2487.7±901.2	2534.2±936.8	<0.0001
BMI (kg/m²)	24.2±3.61	24.4±3.6	24.56±3.65	24.36±3.71	<0.0001
WC (cm)	81.69±10.01	82.94±10.2	84.07±10.24	84.3±10.45	<0.0001
SBP (mmHg)	129.81±18.99	129.54±18.55	130.05±18.02	130.99±18.5	<0.0001
DBP (mmHg)	78.49±11.42	79.08±11.64	79.67±11.61	80.83±12.02	<0.0001
FPG (mmol/L)	5.32±1.37	5.29±1.36	5.36±1.53	5.35±1.47	0.063
TC (mmol/L)	4.7±0.94	4.72±0.98	4.74±0.96	4.74±0.93	0.007
TG (mmol/L)	1.47±1.13	1.55±1.18	1.59±1.27	1.55±1.22	<0.0001
HDL-C (mmol/L)	1.29±0.32	1.25±0.32	1.26±0.33	1.28±0.35	0.073
LDL-C (mmol/L)	2.91±0.83	2.95±0.86	2.96±0.85	2.94±0.83	0.012

*Abbreviations: E: dietary energy; BMI: body mass index; WC: waist circumference; SBP: systolic blood pressure; DBP: diastolic blood pressure; FPG: fasting plasma glucose; TC: total cholesterol; TG: triglyceride; HDL-C: high-density lipoprotein cholesterol; LDL-C: low-density lipoprotein cholesterol.

Table S2. Subgroup analysis of associations between MetS and habitual tea consumption

Subgroup	*n* of overall (%)	OR (95% CI)*				*p* for interaction
		None	1~2 cups/day	3~4 cups/day	Over 5 cups/day	
Age						0.067
18~<30	5510 (12.59%)	Ref.	1.056 (0.792, 1.409)	0.9 (0.594, 1.364)	0.804 (0.536, 1.206)	
30~<45	13,711 (31.33%)	Ref.	0.943 (0.817, 1.089)	0.893 (0.755, 1.056)	0.818 (0.699, 0.958)	
45~59	24,536 (56.07%)	Ref.	0.99 (0.895, 1.094)	0.924 (0.827, 1.033)	0.864 (0.785, 0.951)	
Physical activity						0.6346
Low	10,178 (23.26%)	Ref.	0.855 (0.723, 1.01)	0.859 (0.713, 1.034)	0.848 (0.721, 0.996)	
Medium	10,743 (24.55%)	Ref.	0.966 (0.825, 1.131)	0.873 (0.728, 1.047)	0.802 (0.684, 0.94)	
High	22,836 (52.19%)	Ref.	1.051 (0.942, 1.173)	0.948 (0.835, 1.075)	0.842 (0.752, 0.943)	
Sleep duration (h)						0.7935
<7	7533 (17.22%)	Ref.	0.886 (0.731, 1.072)	0.969 (0.779, 1.205)	0.809 (0.672, 0.974)	
7~8	27,144 (62.03%)	Ref.	1.023 (0.926, 1.129)	0.901 (0.806, 1.009)	0.837 (0.756, 0.928)	
⩾9	9080 (20.75%)	Ref.	0.953 (0.794, 1.144)	0.886 (0.715, 1.097)	0.854 (0.715, 1.02)	
Current smoker						0.0451
No	32,376 (73.99%)	Ref.	0.949 (0.864, 1.041)	0.873 (0.78, 0.978)	0.782 (0.704, 0.869)	
Yes	11,381 (26.01%)	Ref.	1.116 (0.96, 1.298)	1.004 (0.86, 1.171)	0.938 (0.825, 1.066)	
Excessive drinking						0.0359
No	39,431 (90.11%)	Ref.	0.965 (0.887, 1.05)	0.888 (0.806, 0.979)	0.799 (0.731, 0.873)	
Yes	4326 (9.89%)	Ref.	1.16 (0.914, 1.473)	1.084 (0.848, 1.386)	1.056 (0.872, 1.279)	

*Adjusted (corresponding stratified variable was not included) for gender, age, BMI, living area, education level, income, marital status, current smoker (Yes/No), excessive drinking (Yes/No), physical activity, sleeping duration, sedentary behavior, medical examination within one year (Yes/No), DASH-diet score, daily energy intake (Kcal/day), and family history for hypertension and diabetes; Indicated by odds ratio (OR) and 95% confidence interval (CI).
